# Clinical Reality and Treatment for Local Recurrence of Rectal Cancer: A Single-Center Retrospective Study

**DOI:** 10.3390/medicina57030286

**Published:** 2021-03-19

**Authors:** Michał Jankowski, Manuela Las-Jankowska, Andrzej Rutkowski, Dariusz Bała, Dorian Wiśniewski, Karol Tkaczyński, Witold Kowalski, Iwona Głowacka-Mrotek, Wojciech Zegarski

**Affiliations:** 1 Department of Surgical Oncology, Ludwik Rydygier’s Collegium Medicum, Bydgoszcz, Nicolaus Copernicus University, 85-067 Torun, Poland; manuela.las@cm.umk.pl (M.L.-J.); baldar@cm.umk.pl (D.B.); wiciukow@interia.pl (W.K.); zegarskiw@cm.umk.pl (W.Z.); 2 Department of Surgical Oncology, Oncology Center—Prof Franciszek Łukaszczyk Memorial Hospital, 85-796 Bydgoszcz, Poland; dorian.wisniewski89@gmail.com (D.W.); karoltkaczynski@gmail.com (K.T.); 3 Department of Clinical Oncology, Oncology Center—Prof Franciszek Łukaszczyk Memorial Hospital, 85-796 Bydgoszcz, Poland; 4 Department of Gastroenterological Oncology, M. Skłodowska-Curie Memorial Cancer Centre, 02-781 Warsaw, Poland; andrzej.rutkowski@pib-nio.pl; 5 Department of Rehabilitation, Ludwik Rydygier’s Collegium Medicum, Bydgoszcz, Nicolaus Copernicus University, 85-067 Torun, Poland; iwona.glowacka@cm.umk.pl

**Keywords:** rectal cancer, local recurrence, surgery, resection, radiotherapy, radiochemotherapy

## Abstract

*Background and Objectives:* Despite advances in treatment, local recurrence remains a great concern in patients with rectal cancer. The aim of this study was to investigate the incidence and risk factors of local recurrence of rectal cancer in our single center over a 7-year-period. *Materials and Methods:* Patients with stage I-III rectal cancer were treated with curative intent. The necessity for radiotherapy and chemotherapy was determined before surgery and/or postoperative histopathological results. *Results:* Of 365 rectal cancer patients, 76 (20.8%) developed recurrent disease. In total, 27 (7.4%) patients presented with a local tumor recurrence (isolated in 40.7% of cases). Radiotherapy was performed in 296 (81.1%) patients. The most often used schema was 5 × 5 Gy followed by immediate surgery (*n* = 214, 58.6%). Local recurrence occurred less frequently in patients treated with 5 × 5 Gy radiotherapy followed by surgery (*n* = 9, 4%). Surgical procedures of relapses were performed in 12 patients, six of whom were operated with radical intent. Only two (7.4%) patients lived more than 5 years after local recurrence treatment. The incidence of local recurrence was associated with primary tumor distal location and worse prognosis. The median overall survival of patients after local recurrence treatment was 19 months. *Conclusions:* Individualized rectal cancer patient selection and systematic treatment algorithms should be used clinical practice to minimize likelihood of relapse. 5 × 5 Gy radiotherapy followed by immediate surgery allows good local control in resectable cT2N+/cT3N0 patients. Radical resection of isolated local recurrence offers the best chances of cure.

## 1. Introduction

It is estimated that in 2020, rectal cancer with the number of 732,210 cases is going to be the 8th most common cancer in the world, responsible for 339,022 deaths [1]. The number of colorectal cancer cases is increasing in Europe and is related to the western lifestyle [2]. Poland is one of the countries with an increasing number of colorectal cancer cases [3]. In 2018, in Poland, 6448 new cases were diagnosed with rectal cancer, and 3347 people died thereof [4].

Local recurrence is commonly perceived as a failure of surgical treatment due to inadequate or poor surgical techniques [5], and therefore, it still remains a challenge in modern surgery. The local recurrence rates decreased from 50% to 20–30% after introduction of abdominoperineal resection at the beginning of the 20th century [6]. Moreover, introduction of many improvements in surgical treatment, such as total mesorectal excision (TME), standardized surgery, radial margin identification, and preoperative radiotherapy/radiochemotherapy, was essential to improve outcomes of oncological rectal cancer surgery and to decrease the local recurrence rate to 4–8% [7,8,9,10,11,12,13,14].

Modern management is adjusted to individual clinical cases it is based on preoperative diagnostics and is a result of conclusions from the publications of the last 3 decades. In recent years, there has been a tendency to increase the interval between preoperative radiotherapy and surgery, which is aimed to reduce the number of surgical complications [15] or even allow for the diagnosis of complete clinical response [16].

The local recurrence rate is still associated with decreased overall survival. Diagnosis and treatment of rectal cancer are a difficult clinical problem, with a relatively rare occurrence. Due to the multifactorial etiology, the problem of local recurrence of rectal cancer can be considered only in the context of general treatment and follow-up procedures.

The aim of this single-center retrospective study was to analyze patterns, incidence, and risk factors of local recurrence in Polish patients treated for rectal cancer by multimodal therapy and using the TME approach.

## 2. Materials and Methods

### 2.1. Patients

Data of 417 adult patients who underwent TME for primary rectal cancer at Prof Franciszek Łukaszczyk Memorial Hospital Oncology Center between January 2001 and December 2008 and who were classified according to the 7th edition of the AJCC/UICC TNM staging system were retrospectively collected [17]. Patients with stage IV and/or unresectable disease were excluded, leaving a total of 365 patients who underwent curative resection. All procedures were performed by an experienced operating team. Postoperative circumferential radial margin data was not collected.

The treatments and research were in accordance with the ethical standards set by the Declaration of Helsinki and research was approved by an independent Ethical Committee of the Collegium Medicum Nicolaus Copernicus University (No. KB533/2016, approved on 29 January 2020).

### 2.2. Treatment Planning, Diagnostic Examinations 

The pretreatment cTNM classification based on diagnostic examinations was established for all patients: colonoscopy with pathological verification, chest X-ray or computed tomography (CT)-scan, abdomen ultrasound or CT-scan, pelvic magnetic resonance imaging (MRI) or CT-scan clinical assessment of mobility.

Based on diagnostic examination, primary tumor resectability was assessed. Highly located tumors (above 12 cm from anal verge) and/or cancer classified as cT1 or cT2 were not treated with preoperative radiotherapy.

### 2.3. Treatment

A total of 365 patients had TME. A rectal anterior resection was performed in 228 patients (62.5%), an abdominoperineal resection in 129 (35.3%), and Hartmann’s resection in eight (2.2%; Table 1). 

In 214 (58.6%) patients with resectable and cT3 and/or cN0-N+ tumors, preoperative short-course (5 × 5 Gy) radiotherapy followed by TME surgery within one week was performed. 

Fifty patients with unresectable tumor or tumors with questionable resectability received preoperative radiotherapy at dose of 1.8–2 Gy in 25–28 fractions and boost with the further 5.4 Gy to the recommended dose of 50.4–54 Gy. Of these patients, 20 received preoperative radiotherapy with concurrent chemotherapy (2 courses of 5-fluorouracil [400 mg/m^2^] and leucovorin [400 mg/m^2^]) for better local response and tumor downstaging in case of no contraindications.

Twenty-six patients with poor prognostic factors (pT3-4 and/or N+) and lack of preoperative radiotherapy received postoperative radiotherapy at dose of 1.8 Gy per fraction followed by a boost of 5.4 Gy to a total dose of 50.4–54 Gy. 

Seventy-one (19.4%) patients did not receive perioperative treatment in case of contraindications for radiotherapy (e.g., cT1-2N0, previous radiotherapy in pelvic region, lack of consent for the procedure). 

Seventy-nine (21.6%) patients with stage II and III disease (e.g., grade 3, tumor perforation, lymphovascular, or perineural invasion) received 6–8 courses of postoperative concurrent chemotherapy.

### 2.4. Follow-Up

The minimum follow-up period for survivors was 60 months (range 60-155) after primary surgery. During observation, standard diagnostic tools were used (carcinoembryonic antigen level and clinical examination between 3–6 months, abdomen ultrasound or CT-scan, pelvic MRI or CT-scan, endoscopy with biopsy).

Local recurrence was defined as evidence of at least one of principal (pathologic verification, palpation examination, pelvic bones infiltration, PET CT [+]) criterion and one of secondary (tumor progression by CT, MRI; infiltration of adjacent organs; increase of markers; typical changes by CT, USG, MRI) criterion.

### 2.5. Local Recurrence

Recurrence location was classified into one of the following 4 subsites: central (anastomosis, rectal stump, and perineal), anterior (vagina and bladder), posterior (presacral space), and lateral (pelvic side wall).

### 2.6. Statistical Analysis

Statistical evaluation was performed through univariate (chi-squared test, “t” test, Snedecor’s F distribution, Mann–Whitney U test, or Cox’s test was used when appropriate) and multivariate analyses (Cox’s regression model) using the STATISTICA software. To compare the difference between factors in surgical procedure, distance from the anal verge, and clinical stage, we performed the chi-squared test with correction for multiple comparisons and the Dunn–Bonferroni post-hoc test. A *p*-value < 0.05 was set as the statistical significance level.

## 3. Results

### 3.1. Patients

Of the 365 analyzed patients, 166 (45.5%) were female. The median patient age at diagnosis was 64 years (range 33–90). The most prevalent clinical stage was stage III with 153 (41.9%) cases (Table 1). There were 106 (29.0%) patients with yp/pT3N0 and 76 (20.8%) with yp/pT2N0 on the final pathology. A total of 358 (98.1%) patients had R0 and seven (1.9%) had R1 resection. In R1 group, no local recurrence was observed.

The incidence of recurrence (local and distant) was 20.8% (*n* = 76). Local recurrence was confirmed in 27 (7.4%) patients (Table 1); 11 (41.0%) and 16 (59.0%) patients had isolated and combined recurrence, respectively. In the latter group, three (18.7%) had local recurrence, three (18.7%) had distant recurrence first, and ten (62.5%) had simultaneous local and distant recurrence. Central (*n* = 8, 29.6%) recurrence was the most common type of local recurrence among the whole group (*n* = 27), similarly distributed in particular pelvic areas (Table 2). In patients with isolated local recurrence, most common was anastomotic location (4/11, 36.6%). Of the all five patients with local recurrent anastomosis, three did not receive perioperative radiotherapy.

Local recurrence was more often observed in female patients (*p* > 0.05) and in those with tumor localized close to the anal verge (0–6 cm, *p* > 0.05; Table 1).

### 3.2. Local Recurrence after Treatment for Rectal Cancer

Local recurrence rate was similar in all types of surgical resections. The rate of local recurrences was the lowest for patients operated after short-term (5 × 5 Gy) preoperative radiotherapy compared with standard radiotherapy (50.4 Gy) or radiochemotherapy (4% vs. 10% or 20%; Table 3). However, these groups included patients with different clinical stages. The incidence of local recurrence was the highest in patients with advanced p/ypT4N2 tumors (4/7, 57.1%) and the lowest in those with p/ypT2N0 tumors (3/76, 3.9%; Table 3). Interestingly, two patients with p/ypT2N0 tumors had intraoperative complications (bleeding, iatrogenic ureter injury), and one had isolated anastomosis local recurrence 4 months after primary surgery.

The recurrence rate in group of patients without radiotherapy was 11.2% (8/71); patients with pT3N2 (1/4, 25.0%) and pT2N0 (3/20, 15.0%) had the highest rate of local recurrence, and in those with pT4N2 tumors, local recurrence was present in all cases (100%).

Among 264 patients with preoperative radiotherapy, pathological complete response was observed in seven cases; in two after short preoperative radiotherapy (out of 214, 0.9%) and in five after radiotherapy/chemoradiotherapy (out of 50, 10.0%). In this letter group of patients one local recurrence was reported (Table 3).

### 3.3. Treatment of Local Recurrence

All patients with confirmation of local recurrence were included in the process of qualifying for treatment. Twelve (44.4%) patients with local recurrence underwent surgery; six (22.2%) had radical resection, and six (22.2%) underwent non-radical resection or palliative procedures. Six (22.2%) patients were not treated and remained under observation or symptomatic treatment. In total, three (11.1%) patients received radiotherapy, four (14.8%) chemotherapy, and two (7.4%) radiotherapy with concurrent chemotherapy (Table 4).

In six patients confined to observation or symptomatic treatment (Table 4), distant metastases (all synchronous) with posterior (*n* = 3), perineal (*n* = 2), and anastomotic (*n* = 1) location were confirmed.

### 3.4. Survival after Local Recurrence Incidence

The mean OS of patients after treatment of local recurrence was 25.7 months, with survival time depending on the therapeutic procedures used and type of relapse (Table 4). Among patients with isolated local recurrence at diagnosis and R0 resection (4/27, 14.8%), only two (7.4%) were alive at 5 years following the first curative surgery (*n* = 1: an isolated form of local recurrence, but without symptoms of relapse, *n* = 1: hepatic metastasis after local recurrence incident treated by curative surgery.

Overall survival was slightly better in patients with isolated recurrence than in those with combined recurrence. In the group of patients treated with radiotherapy and chemotherapy, slightly longer OS was observed in patients who underwent palliative surgery (Table 5).

The longest mean OS after treatment of local recurrence was observed in patients with central recurrences (37 months), and the largest differences in survival times were found for patients with anastomosis and perineum recurrent rectal cancer (60.4 months vs. 3.3 months, *p* = 0.05). The long survivors had local recurrence localized in anastomosis. Patients with posterior and lateral located tumors had lower average survival rates compared to those with front side tumors (Table 5).

## 4. Discussion

In our series of patients with rectal cancer, the incidence and risk factors of local recurrence of rectal cancer was investigated. Patients were treated by the same surgeons within the same institution over a 7-year-period.

The analysis of patients with local recurrence only revealed that the use of short-term 5 × 5 Gy preoperative radiotherapy followed by surgery (5–7 days after) led to statistically significant decrease of the local recurrence rate to 4.2% compared with 10–20% in the standard preoperative radiotherapy (50.4 Gy) or radio-chemotherapy groups. These were similar patient populations, except clinical stage. This observation is in concordance with other reports [17,18]. The Dutch Colorectal Cancer Group (the Dutch TME trial) demonstrated that the addition of short-course preoperative radiotherapy to optimal surgery with total mesorectal excision reduced the rate of local recurrence in all primary tumor sites [10]. Other two randomized trials have also demonstrated an advantage for the preoperative irradiation scheme [11,19,20]. It should be noted however that comparison of the effects of perioperative radiotherapy on local recurrence across different studies is difficult due to different treatment regimens, qualification systems for combination therapy, schemes, and types of radiotherapy used [21,22]. In the Dutch TME trial, short-course radiotherapy was particularly effective in preventing anastomotic local recurrence after anterior resection of the rectum [10]. Interestingly, contemporary schemas of combined treatment increasingly recommended to long interval between preoperative radiotherapy and surgery [15,23,24].

In our study, in one of nine local recurrence cases, a recurrent lesion was at the anastomotic line. A high rate of relapses was observed in patients treated with radiotherapy only (50.4 Gy) and in those receiving the radiotherapy and chemotherapy combination prior to surgery. In these patients, due to the tumor progression, preoperative treatment was given in order to downstage primary rectal cancer. Despite clinical and pathological tumor downstaging, the recurrence rate was 20%, with lateral (pelvic side wall) site being the most common location of recurrence. These outcomes are similar to other reports [25].

We estimated from postoperative pTNM that at least 43 patients had indications for perioperative radiotherapy; 40 patients had resectable tumors, which may have been irradiated before surgery as in case of patients with the short course (5 × 5 Gy) radiotherapy. The local recurrence rate was higher in patients without perioperative radiotherapy than in those with the short course radiation treatment (10.0% vs. 4.0%). This observation supports the importance of the perioperative radiotherapy considerations.

One of the most important risk factors for local recurrence is the severity of disease at the time of an initial operations [26,27]. Over half of patients with p/ypT4N2 in our series did not have local recurrence after primary tumor treatment (42.9%). A pathological complete response after preoperative treatment was observed in seven patients; a proportion consistent with others reports [28].

In our analysis, low location of rectal cancer was associated with a greater risk of local recurrence. In the Dutch TME trial, patients with low rectal cancer who underwent curative intent abdominoperineal resection had negative circumferential resection margin, were N+, and had local recurrence rate of 18% [10]. The authors suggested that anatomical structure of this area (the small width of the mesorectum, especially from the front side) might be responsible for these results and proposed wide surgical resection procedure in this setting. Abdominosacral resection may result in fewer local recurrences compared to abdominoperineal procedure [29]. Currently, distance of the tumor to the anal verge defined in the preoperative examination is considered an independent risk factor for recurrence [23,26,30,31,32].

Despite an overall effect of preoperative short-term radiotherapy on local control in patients with clinically resectable rectal cancer in our study, there was no effect on OS (the 5-year OS of 43% after R0 resection). Although not directly comparable, other studies also failed to show an OS benefit, despite an improved rate of local recurrence [20,33].

In our series, local recurrence with metastases was observed in about half of patients, but only one patient survived longer than 5 years. This small survival benefit observed here is in line with previous findings [19,29,33,34,35,36,37]. Interestingly, a patient who was alive at 5 years in our study had oligometastatic disease.

Treatment of local recurrence in patients with rectal cancer should be individualized and reviewed by a multidisciplinary therapeutic team [38,39], include all available therapeutic methods, and be considered in the case of distant metastases. Moreover, a local treatment should, whenever possible, include radiotherapy [23,30]. However, radical surgical excision of local recurrence (resection R0) remains, in the opinion of most specialists, the best option for successful treatment of local recurrence [40,41,42]. The analysis of the Swedish Colorectal Cancer Registry population-based data, reported by Westberg et al., showed that R0 resection is the only effective treatment in patients with locally isolated recurrent rectal cancer and in patients who experienced local recurrence before distant metastases [37]. However, less than one third of all patients included in the study underwent a resection of locally recurrent rectal cancer with curative intent, and of these, only 53% had an R0 resection (which represents 6.5% of the study population). The 5-year overall survival after R0 resection was 43%. In our study, with the same qualification procedure, R0 resection was performed in 75% of patients (6/8), but only two (25.0%) survived for more than 5 years after this treatment, which represents 7.5% of the whole population with local recurrence.

The strength of our study is in the length of follow-up and single center character, which impact to standardized surgery and radiotherapy. However, there are also some limitations. Our work presents a retrospective analysis, and therefore, selection bias might be facilitated and the low number of patients in certain groups might not allow safe statistical conclusions. However, given that the medical records of patients specific to the tumor were properly retained, therefore, the risk of information bias due to loss of records is less and does not affect the reliability of the results. Our results are not revolutionary, and are similar to the results of other publications. However, we would like to underscore that these data and outcomes (e.g., 4.9% LR at 5 × 5 Gy after immediate surgery in resectable cT3/4N0/+M0 cancers) were obtained based on previous recommendations [43,44] and may constitute the basis for discussion on treatment outcomes in contemporary research.

## 5. Conclusions

Despite advances in treatment of rectal cancer and relatively low rates, local recurrence remains therapeutic challenge. Probably, the best way to prevent relapses is to make more precise qualification of patients and to treat the primary disease with combination treatment. Satisfactory results for local recurrence treatment are possible only in the case of R0 resection and in about half of patients with rectal cancer. Further prospective clinical trials are necessary in order to clearly define the impact of multimodal therapeutic strategies in patients with local recurrence of rectal cancer.

## Figures and Tables

**Table 1:** Patient characteristics (*n* = 365).

	No Recurrence	Local Recurrence	*p*
	*N* = 338	*N* = 27	
	*n* (%)	*n* (%)	
Sex			<0.05
Male	192 (56.8)	7 (25.9)	
Female	146 (43.2)	20 (74.1)	
Age, years median/range	64 (33–90)	62 (44–86)	NS
Surgical procedure			
Dixon	213 (63.0)	15 (55.5)	NS
Miles	118 (35.0)	11 (41.0)	
Hartmann’s	7 (2.1)	1 (3.7)	
Re-operation	25 (7.4)	2 (7.4)	NS
Surgical complications	92 (27.2)	8 (29.6)	NS
(30 days postoperative)			
Average tumor size, mm	39.6	39.1	NS
Distance from the anal verge, cm	7 (1–15)	5.5 (1–15)	
˂6	141 (41.7)	17 (63.0)	NS
7–12	146 (43.2)	8 (29.6)	
13–15	21 (6.2)	1 (3.7)	
No data or data incomplete	30	1	
Clinical stage (ypTNM/pTNM)			NS
I	86 (25.4)	3 (11.1)	
II	107 (31.7)	9 (33.3)	
III	139 (41.1)	14 (51.8)	
pCR	6 (1.8)	1 (3.7)	

Abbreviations: NS, not statistically significant; pCR, pathological complete response.

**Table 2:** Local recurrence according to type of treatment.

	Total *N* = 365 *n* (%)	Local Recurrence *N* = 27 *n* (%)	All Recurrences %
No RT	71 (19.4)	8 (29.6)	11
Preoperative RT			
spRT	214 (58.6)	9 (33.3)	4
pCRT	20 (5.5)	4 (14.8)	20
pRT	30 (8.2)	3 (11.1)	10
Postoperative RT			
poRT	26 (7.1)	3 (11.1)	11
Others *	4 (1.1)	0	0

Abbreviations: RT, radiotherapy; spRT, short preoperative radiotherapy (5 × 5 Gy); pCRT, preoperative radiochemotherapy (50.4 Gy); pRT, preoperative radiotherapy (50.4 Gy); poRT, postoperative radiotherapy. * Not completed course of RT or delayed surgery.

**Table 3:** Frequency rate of recurrence for patients with rectal cancer according to ypTNM /pTNM staging and without perioperative radiotherapy.

Stage ypTNM/pTNM	Without Recurrence *N* = 338 *n* (%)	With Local Recurrence *N* = 27 *n* (%)	Total *N* = 365 *n* (%)	% Recurrence	RT/−/ *N* = 71 *n* (%)	W RT/−/ LR− *N* = 63 *n* (%)	RT/−/ LR+ *N* = 8 *n* (%)	% Recurrence RT/−/
T0N0	6 (1.8)	1 (3.7)	7 (1.9)	14.3	0		0	0
T0N1	1 (0.3)	0	1 (0.3)	0	0		0	0
T1N0	14 (4.1)	0	14 (3.8)	0	10 (14.8)		0	0
T2N0	73 (21.6)	3 (11.1)	76 (20.8)	3.9	20 (28.2)	17 (26.9)	3 (37.5)	15
T2N1	21 (6.2)	2 (3.7)	23 (6.3)	8.7	4 (5.6)	4 (63.5)	0	0
T2N2	17 (5.0)	1 (3.7)	18 (4.9)	5.5	3 (4.2)	3 (4.8)	0	0
T3N0	98 (29.0)	8 (29.6)	106 (29.0)	7.5	19 (26.8)	17 (26.9)	2 (25)	10.5
T3N1	52 (15.4)	4 (14.8)	56 (15.3)	7.1	11 (15.5)	10 (15.9)	1 (12.5)	9.0
T3N2	38 (11.2)	2 (7.4)	40 (10.9)	5.0	4 (5.6)	3 (4.8)	1 (12.5)	25.0
T4N0	10 (3.0)	1 (3.7)	11 (3.0)	9.0	1 (1.4)	1 (1.6)	0	0
T4N1	5 (1.5)	1 (3.7)	6 (1.6)	16.7	1 (1.4)	1 (1.6)	0	0
T4N2	3 (1.9)	4 (14.8)	7 (1.9)	57.1	1 (1.4)	1 (1.6)	1 (12.5)	100

Abbreviations: RT/−/, without perioperative radiotherapy; LR−, local recurrence.

**Table 4:** Treatment for locally recurrent rectal cancer according to local recurrence site.

Location	*N* (*)	% (*)		RT/CRT Scheduling		
			No RT	sRT	pCRT/pRT	poRT
			*n* (*)	*n* (*)	*n* (*)	*n* (*)
Central						
Anastomosis	5 (4)	18.5 (36)	3 (2)	1 (1)	1 (1)	-
Rectal stump	1 (1)	3.7 (9)	-		1	-
Perineum	3 (2)	11.1 (18)	1	2 (2)		-
Front						
Vagina	4 (1)	14.8 (9)	2	2	-	-
Bladder	1 (0)	3.7 (0)	-	1	-	-
Posterior						
Presacral space	7 (3)	25.9 (27)	2 (1)	2 (1)	1	2 (1)
Side						
Pelvic sidewall	6 (2)	22.2 (18)	-	1	4 (2)	1
Total	27 (11)	100 (100)	8 (3)	9 (4)	8 (2)	2 (1)

* Isolated local-regional recurrence. Abbreviations: RT, radiotherapy; sRT, preoperative radiotherapy (5 × 5 Gy); pCRT, preoperative radiochemotherapy (50.4 Gy); pRT, preoperative radiotherapy (50.4 Gy); poRT, postoperative radiotherapy.

**Table 5:** Overall survival in patients with local recurrence according to treatment procedures, type of recurrence and site.

	*n*	Median OS after PT	*p*	Median OS after LRT	*p*
		Months (Range)		Months (Range)	
Type of recurrence					
Isolated	11	53 (9–161)	0.416	21 (2–119)	0.779
Combined	16	45 (9–149)		18 (2–116)	
All	27	47 (9–161)		19 (2–119)	
Site					
Combined + isolated	27				
Central	9	37 (9–161)		21 (1–119)	
Anastomotic	5	65 (25–161)		23 (21–119)	
Perineal	3	11 (9–19)		2 (2–6)	
Anterior	5	48 (38–84)		25 (18–59)	
Posterior	7	47 (20–74)		17 (3–40)	
Lateral	6	42 (24–110)		13 (7–36)	
Isolated	11				
Central	5	25 (9–161)		21 (2–119)	
Anastomotic	3	53 (25–161)		23 (21–119)	
Perineal	2	14 (9–19)		4 (2–6)	
Anterior	1	84 (84)		59 (59)	
Posterior	3	72 (34–74)		17 (3–22)	
Lateral	2	67 (24–110)		21 (7–36)	
Procedures					
Palliative surgical procedures	4	42 (19–65)		14 (7–24)	
Non radical resection	2	42 (19–65)		14 (6–23)	
Radical resection	6	48 (25–161)		34 (15–119)	
RT/CT	9	56 (34–110)		19 (3–59)	
Observation	6	34 (9–72)		4 (2–23)	

Abbreviations: OS, overall survival; PT, primary treatment; LRT, local recurrence treatment; RT, radiotherapy; CT, chemotherapy.

## Data Availability

The datasets generated during and/or analysed Turing the current study are available from the corresponding author on responsable request.
